# Nutritional Controlled Preparation and Administration of Different Tomato Purées Indicate Increase of β-Carotene and Lycopene Isoforms, and of Antioxidant Potential in Human Blood Bioavailability: A Pilot Study

**DOI:** 10.3390/nu13041336

**Published:** 2021-04-17

**Authors:** Daniela Vitucci, Angela Amoresano, Marcella Nunziato, Simona Muoio, Andreina Alfieri, Giovannangelo Oriani, Luca Scalfi, Luigi Frusciante, Maria Manuela Rigano, Piero Pucci, Luigi Fontana, Pasqualina Buono, Francesco Salvatore

**Affiliations:** 1 CEINGE-Biotecnologie Avanzate, Via G. Salvatore, 486, 80145 Naples, Italy; vitucci@ceinge.unina.it (D.V.); nunziato@ceinge.unina.it (M.N.); andreina.alfieri@uniparthenope.it (A.A.); oriani@ceinge.unina.it (G.O.); pucci@unina.it (P.P.); 2 Department of Chemical Sciences, University of Naples “Federico II”, via Cinthia, 80126 Naples, Italy; angamor@unina.it; 3 Department of Molecular Medicine and Medical Biotechnologies, University of Naples “Federico II”, via Sergio Pansini 5, 80131 Naples, Italy; 4 Department of Public Health, School of Medicine, University of Naples “Federico II”, 80131 Naples, Italy; smonci@gmail.com; 5 Department of Human Movement Sciences and Wellbeing, University of Naples “Parthenope”, Via Medina, 40, 80133 Naples, Italy; 6 Institute of Internal Medicine and Metabolic Diseases, Medical School, University of Naples, Federico II, 80131 Naples, Italy; scalfi@unina.it; 7 Department of Agricultural Sciences, University of Naples ‘Federico II’, Via Università 100, Portici, 80055 Naples, Italy; fruscian@unina.it (L.F.); mariamanuela.rigano@unina.it (M.M.R.); 8 Charles Perkins Center, Faculty of Medicine and Health, University of Sydney, Science Rd, Camperdown, Sydney, NSW 2050, Australia; luigi.fontana@sydney.edu.au; 9 Department of Endocrinology, Royal Prince Alfred Hospital, 50 Missenden Rd, Camperdown, Sydney, NSW 2050, Australia; 10 Department of Clinical and Experimental Sciences, Brescia University, Viale Europa, 11, 25123 Brescia, Italy

**Keywords:** tomato sauces, lycopene, human health, antioxidant power, tomato purée

## Abstract

The isoforms of lycopene, carotenoids, and their derivatives including precursors of vitamin A are compounds relevant for preventing chronic degenerative diseases such as cardiovascular diseases and cancer. Tomatoes are a major source of these compounds. However, cooking and successive metabolic processes determine the bioavailability of tomatoes in human nutrition. To evaluate the effect of acute/chronic cooking procedures on the bioavailability of lycopene and carotene isoforms in human plasma, we measured the blood levels of these compounds and of the serum antioxidant potential in volunteers after a meal containing two different types of tomato sauce (*rustic* or *strained*). Using a randomized cross-over administration design, healthy volunteers were studied, and the above indicated compounds were determined by HPLC. The results indicate an increased bioavailability of the estimated compounds and of the serum antioxidant potential with both types of tomato purée and the subsequently derived sauces (the increase was greater with *strained* purée). This study sheds light on the content of nutrient precursors of vitamin A and other antioxidant compounds derived from tomatoes cooked with different strategies. Lastly, our study indicates that strained purée should be preferred over rustic purée.

## 1. Introduction

Tomato (*Solanum lycopersicum* L.) is an important component of the Mediterranean diet. It is a rich source of such micronutrients as carotenoids, vitamin C, folate, and flavonoids that are known to promote human health and wellbeing [1,2,3,4]. Carotenoids are a class of compounds containing approximately 600 highly hydrophobic bioactive molecules that are represented in plant *taxa.* They also serve as pigments, being responsible for the varied and vivid colors present in nature [5,6].

Among carotenoids, lycopene (C_40_H_56_) is one of the six essential pigments (β-carotene, α-carotene, γ-carotene, lycopene, zeaxanthin, lutein, and others) discovered starting in 1876, that give the red color to a variety of fruits and vegetables including tomato, watermelon, grapes, apricots, and pink guava [7,8]. Lycopene is abundant in tomatoes, ranging from 8.8 to 42.0 µg/g fresh weight fruit/vegetable [9] corresponding up to 90% of the total carotenoid content [10,11].

This pigment seems to protect against chronic degenerative diseases by mitigating oxidative damage [12]. The lycopene’s antioxidant properties are due to its highly unsaturated composition, which consists of an open straight hydrocarbon chain of 11 conjugated and two unconjugated double bonds [13]. These conjugated double bonds are principally responsible for the characteristic deep red color and for their antioxidant activity [14]. The anti-free radical effect of lycopene in reducing cardiovascular disease was reported to be double that of β-carotene [15,16,17,18,19]. Furthermore, carotenoids in general, and lycopene in particular, may act as anti-carcinogenic agents [20,21]. Indeed, lycopene dietary intake and blood concentration have been negatively associated with cancer, particularly prostate cancer [22,23,24,25,26]. Moreover, epidemiological trials indicate that lycopene also protects against neurodegenerative and such other chronic diseases as asthma, hypertension, osteoporosis, and others [18,24,27,28,29]. Lycopene is a hydrophobic molecule soluble in fat: in fact, its bioavailability has been estimated at about 23% when pure lycopene was mixed with olive oil [30], and at about 5% when it was ingested as tomato juice [31]. The potential availability of antioxidants after the digestion process is critical, and studies reported a partial loss of the bioactive compounds after digestion [32]. Indeed, the bioaccessibility of carotenoids can be affected by many factors, including the food matrix, processing, and cooking methods, and the interactions that occur during digestion and absorption with other dietary compounds, such as fibers, lipids, phytosterols, and other carotenoids [33,34]. Among dietary factors, heat and mechanical treatment of foods and the presence of fat in a meal appear to be critical for carotenoid bioaccessibility and plasmatic bioavailability in vivo [1,35,36]. The carotenoid structure also plays an important role in plasma bioavailability. Lycopene exists in a variety of geometric isomers, including all-*trans*, mono-*cis*, and poly-*cis* forms. The all-*trans* isomer of lycopene is predominant in fresh tomatoes and different various tomato-based foods, ranging from 35 to 96% of total lycopene; it is also the most thermodynamically stable form [37]. During tomato processing and storage, lycopene can undergo *trans*-to-*cis* isomerization. On the other hand, lycopene *cis*-isomers contribute more than 50% to the total lycopene in human serum and tissue [38,39].

Since the potential plasmatic availability of antioxidants after the digestion process is essential, in order to exert beneficial effects on health, in the present pilot study, we investigated the effects of different cooking times on lycopene availability in two commercial tomato purées of different textures, *rustic* and *strained*, and compared the post-prandial bioavailability of lycopene isomers and carotene in the plasma of healthy volunteers after an acute (single-dose) and a chronic (five days) test-meal consumption, containing the sauces obtained from the purées.

In particular, our aims were as follows: (a) to assess the bioaccessibility of lycopene isomers in sauces made with two different tomato purées, after increased boiling time, with and without extra-virgin olive oil (evo); (b) to test the bioavailability of *trans*- and *cis*- isoforms of lycopene and carotene in plasma in healthy volunteers after test-meal consumptions (acute phase and chronic phase), containing sauces obtained from the two different tomato purées; and (c) to test total biological antioxidant potential (BAP) in serum of healthy volunteers after consumption of chronic the test-meal consumption (chronic phase).

## 2. Materials and Methods

### 2.1. Subjects

Twelve healthy, non-smoking volunteers (three men and nine women aged 25–48 years were recruited for the study at CEINGE Biotecnologie Avanzate of Naples. Eligibility was based on a screening test for normal blood lipid profiles and health nutrition and lifestyle questionnaires (EPIC and SF36) [40]. Exclusion criteria were any clinical, behavioral, or psychological condition that could interfere with a correct participation of the subject in the study and compromise that could impair the compliance: intolerance and/or tomato puree allergies or allergies of and other foods allergies in the test meal, use of drugs that can interfere with the parameters measured in the study (i.e., NSAIDs, cortisones or antibiotics), frequent alcohol consumption, special diet regimens (e.g., vegetarian, vegan, low-calorie diets, etc.), metabolic, renal, gastrointestinal, hepatic diseases or anemia; use of multivitamin supplements containing carotenoids or lycopene, state of pregnancy or breastfeeding.

The study was conducted according to the Declaration of Helsinki and approved by the Ethics Committee of the University of Naples Federico II (protocol number 266/17), and written informed consent was obtained from participants before starting the experimental protocol (Ethics Committee documentation, annex 4).

### 2.2. Nutritional Characteristics of Tomato Purées

Two tomato purées, commercialized as *rustic tomatoes* (more dense and compact) and *strained* tomatoes (less compact and filtered to be less granular) were kindly provided by Bioitalia, Area PIP Sarno, Salerno, Italy, and used in this study. *Rustic* tomatoes *purée* has the following nutritional values per 100 g of product: calories (32 kcal), fats (0.1 g), saturated fats (0.0 g), total carbohydrates (6.0 g) of which sugars (5.1 g) and dietary fiber (1.1 g), proteins (1.3 g), salt (0.3 g); *strained* tomatoes purée has the following nutritional values per 100 g of product: calories (26 kcal), fats (0.2 g), saturated fats (0.0 g), total carbohydrates (4.6 g) of which sugars (2.4 g) and dietary fiber (0.0 g), proteins (1.5 g), sodium (0.5 g). To the aim of verifying the bioavailability of compounds present in these two purées, we decided to use a more dense versus a less compact purée deriving from the same company to better evaluate this commercial feature.

### 2.3. Test Meal Preparation and Composition

The two tomato sauces used for the test meal were prepared each in a single solution according to standardized methods. and completely comparable between the two sauces. The initial volume of puree to be processed was calculated based on obtaining 200 g of uncooked tomato sauce for each subject. The tomato sauces were prepared from *rustic tomato* and *strained tomato* purées as follows: 50 g of extra virgin olive oil per liter of tomato sauce, salt, and basil. Purées were boiled for 30 min to obtain a volume reduction of 25%. At the end of the cooking, single-serve portions of 150 g of sauce were prepared. Both tomato purées were cooked the day before the test meal, portioned, and frozen according to the number of volunteers, to be used in the acute and chronic meal tests. The portions prepared for the chronic experimentation were frozen at −20 °C and stored until the time of the consumption; then, they were thawed and heated in a microwave before the test meal. The test meal consisted of tomato sauce (150 g) served with 5 slices of white bread (120 g) and 500 mL bottled water, about half of which was to be consumed during the meal. The energy value of the meal was 474 kcal with *rustic* tomato purée and 462 kcal with *strained* tomato purée.

### 2.4. Experimental Design

**Figure 1:**
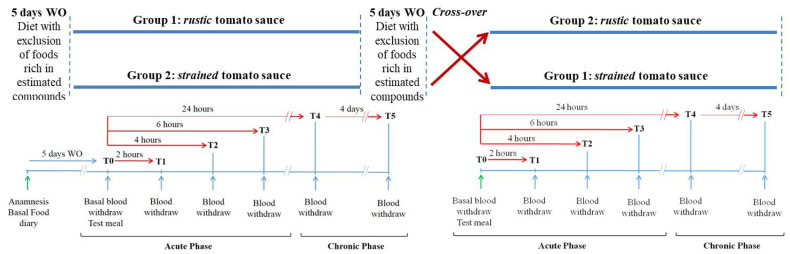
Experimental plan shows a randomized cross-over design. Five days of washout (WO) precede baseline blood withdrawal (T0); blood samples collected at 2, 4, 6, and 24 h (T1, T2, T3 and T4) following test meal consumption; blood withdrawal after four more days of chronic consumption of tomato sauce (T5). *Strained* tomato sauce and *rustic* tomato sauce are sauces prepared with tomato purèe + dressing + 30 min boiling (see details under Methods section).

Volunteers were enrolled at the site of the experimentation (University of Naples, Federico II) and were instructed to avoid any foods with more than 0.1 mg/100 g of lycopene, according to the USDA-Nutrition Coordinating Centre and National Cancer Institute Nutrient Database (tomatoes and derivatives, pumpkin, water melon, red fruits, etc.) in the 5 days preceding each experimental phase as a washout period in order to avoid carry-over of the examined compounds from previous meals [41]. After the washout period (five days), volunteers started the acute phase of experimental design (see Figure 1). Tomato sauces were administrated in a randomized *cross-over* design, each subject acting as his/her own control. Subjects arrived at the University of Naples, Federico II after a 12-h overnight fasting. After a baseline blood collection (T0), at 9.00 A.M., subjects consumed test meal in about 20 min (called acute-phase, see Figure 1). Then, additional blood samples were collected at 2, 4, 6, and 24 h (T1, T2, T3, and T4) following the test meal consumption. Subject consumed a light dinner not containing tomato or other foods rich in carotenoids. For the 4 days following the test meal, volunteers were instructed to lead a habitual lifestyle similar to that led in the days preceding the experimental sessions. In these 4 days, the subjects consumed the same quantity of tomato sauce once a day (portioned and supplied to the subjects, called chronic phase, see Figure 1) at home, and the morning of the fifth day, the fasting blood sampling was repeated (T5). Then, subjects left the clinic, continued their washout diet for the next 5 days, and returned to the clinic to consume the test meal similar to the first 5 days but with the tomato sauce that they did not consume on their first test meal intake (Figure 1).

### 2.5. Blood Sampling and Analysis

Blood samples, drawn from a forearm vein into BD Vacutainer^®^ EDTA tubes and BD Vacutainer^®^ Plus Plastic Serum Tubes for serum collection (BD and Co., Franklin Lakes, NJ, USA), were immediately centrifuged at 1250× *g* for 10 min at 4 °C (Centrifuge 5415, Eppendorf, Hamburg, Germany). Then, plasma and serum were aliquoted in 500 µL of volume in tubes and immediately stored at −80 °C until use.

### 2.6. Carotenoid and Lycopene Extraction and Estimation

Carotenoids extraction was carried out on both tomato sauces and blood samples. To each sample, a total of 0.8 mL water, 1.6 mL methanol, and 3.2 mL hexane (0.025% butylated hydroxy toluene—BHT) were added to each sample; then, the vials were shaken for 1 min, and hexane was added (2 mL). The upper hexane phase was transferred to a clean glass vial, and dichloromethane (CH_2_Cl_2_, 2 mL) was added to the remaining sample. The sample was again vortexed (1 min) and then centrifuged for 2 min at 3000 rpm (Jouan MR1822, ThermoFisher Scientific, Walham, MA, USA) to induce a clean phase separation. The lower dichloromethane phase was removed and pooled with the hexane extract and dried under a stream of argon at 30 °C in the dark. Then, the residue was diluted in 1 mL of hexane–CH_2_Cl_2_ (1:1, *v*/*v*) evaporated to dryness with N_2_. Dried extracts were stored at −20 °C, and 100 μL of the solution was used for the HPLC analysis.

### 2.7. HPLC Analysis

Tomato and blood extracts were re-dissolved in 50 μL MTBE, to which 50 μL of MeOH was added, and samples were briefly sonicated (<5 s) in a sonication bath for dissolution. Samples were analyzed using an HP1100 (Agilent Technologies, Santa Clara, CA, USA) HPLC system equipped with a photodiode array detector. Chromatography was carried out using a Supelcosil LC-18 analytical column (3 μm, 150 × 4.6 mm i.d, Supelco, Bellefonte, PA, USA), the mobile phase consisted of solvent A (80% methanol) and solvent B (acetonitrile, methanol and tetrahydrofuran 70:25:5, % *v*/*v*) and was delivered at flow rate of 1.0 mL/min. Prior to use, the mobile phase was degassed by sonication. The wavelengths were set at 450 nm (β-carotene), and 471 nm (lycopene).

The HPLC separation was performed by using the following gradient: beginning at 40% B, increasing linearly to 90% B over 12 min, holding at 80% B for 1 min, returning to 40% B over 3 min. The method designed was able to separate 5-5-*cis*-lycopene (which elutes just after the all-trans configuration on a C18 column) from β-carotene and all-trans-lycopene, as well as other *cis*-isomers (which elute prior to the all-trans configuration). The retention times were at 6.1 min for β-carotene, 7.0 min for all-trans lycopene, and 7.2 for 5-5-*cis*-lycopene. Stock solutions of lycopene and carotene for generating standard curves were prepared by dissolving 10 mg of each compound in methanol and THF (50:50, %, *v*/*v*) to yield concentrations of 500 μg/mL. To establish the range of linearity between carotenoid concentration and detector response, the standard concentrations of 0.5, 1.5, 10, 25, and 100 μg/mL were used. Then, three replicates from the same sample of analyte were measured. Carotenoids were quantified using external calibration curves generated from authentic standards by integrating the peak areas at 450 nm (β-carotene) and 471 nm (lycopene).

### 2.8. Biological Antioxidant Potential Test

The test was performed on serum. Sera collected and stored at −20 °C were thawed in ice and analyzed for the BAP test according to the DIACRON Labs S.r.l. protocol [42]. The BAP test is based on the ability of a colored solution, containing ferric ions (Fe^3+^) adequately bound to a special chromogenic substrate, to decolor when Fe^3+^ ions are reduced to ferrous ions (Fe^2+^) as well as it can be observed by adding a reducing system, e.g., serum. On the basis of the protocol, we had dissolved serum samples in a colored solution that was derived by the mixing of ferric chloride (R_2_ reagent) as a source of ferric ions and thiocyanate-derived compound (R_1_ reagent) as a special chromogenic substrate. After 5 min incubation, the solution changed color, and the intensity was directly proportional to the ability of serum to reduce ferric ions. The intensity of decoloration was assessed photometrically, and the amount of reduced ferric ions was adequately calculated. The reducing ability represents the antioxidant power and is related to the tested substrate (ferric ions). This test was used to measure the components of the antioxidant plasma barrier, such as scavengers.

### 2.9. Statistical Analysis

This study was conducted using a randomized *cross-over* design in which participants were randomly assigned into an experimental group or a control group (*rustic* or *strained* tomato sauces), and it is a longitudinal study in which subjects receive a sequence of different treatments after a washout period; thus, each subject acts as his/her own control.

Given the study design, the only expected difference between the control and experimental groups in this randomized controlled trial is the outcome variable being studied. The reduced sample size is balanced by the *cross-over* design of the study.

The ANOVA test was carried out to compare the postprandial plasma concentration of *trans*- and *cis*- lycopene expressed as µg/mL in each time (T1, T2, T3, T4) between the two tomato sauces. Data were analyzed using the Statview statistical software (version 5.0.1.0; SAS Institute). A mixed factorial design with repeated measures ANOVA was used to test the fixed factors of meal and meal × digestion phase, on *trans*- and *cis*-lycopene level (dependent variable). A *p*-value < 0.05 was considered statistically significant. Post hoc analysis was performed according to Bonferroni correction for multiple comparisons to determine statistically significant interactions between meals at the same digestion phase. The area under the curve (AUC) over 24 h was also calculated by trapezoidal approximation [43]. Baseline corrected AUC (nmol × h/L) values are expressed as mean values with their standard errors. A *p*-value < 0.05 was considered significant.

## 3. Results

Ten subjects out of 12 recruited completed the study and consumed both test meals in a randomized *cross-over* design (Figure 1). Unfortunately, two women were obliged to leave the study for personal/familial reasons: therefore, we could not substitute them.

### 3.1. Tomato Samples Treatment and Carotenoids Estimation

*Trans*- and 5-*cis*- lycopene and β-carotene content of two different purées in three different conditions/shapes were determined by HPLC analyses and reported in Table 1. The *strained* tomatoes showed, in basal condition (pureé, i.e., uncooked), higher concentration of both *trans*- and 5-*cis*-lycopene isoforms when compared to *rustic* tomatoes. The heat treatment (10 min boiled purée) increased the concentration of both lycopene isomers and β-carotene. Moreover, the preparation of the sauce, with the addition of evo, preserved or even further increased the content of both lycopene isomers and β-carotene. In particular, the tested compounds were at least 2-fold higher in *strained* tomatoes sauce. The increase in concentration was independent of water content in purées, as witnessed by a similar fixed residue at 105 °C (Table 1).

To assess the effects of cooking time and presence of vehicle (evo) on the enrichment of the estimated compounds in sauces, we carried out a time course of boiling with and without evo. The results indicate that boiling and the addition of olive oil positively affected the *cis*-, *trans*-lycopene, and β-carotene concentration in both sauces in a time-dependent manner. In particular, the effects were similar in the two analyzed sauces, and the effects were maximum in *strained* tomatoes cooked with evo (Table 2).

### 3.2. Lycopene Plasma Content after Tomato Meal Consumption

**Figure 2:**
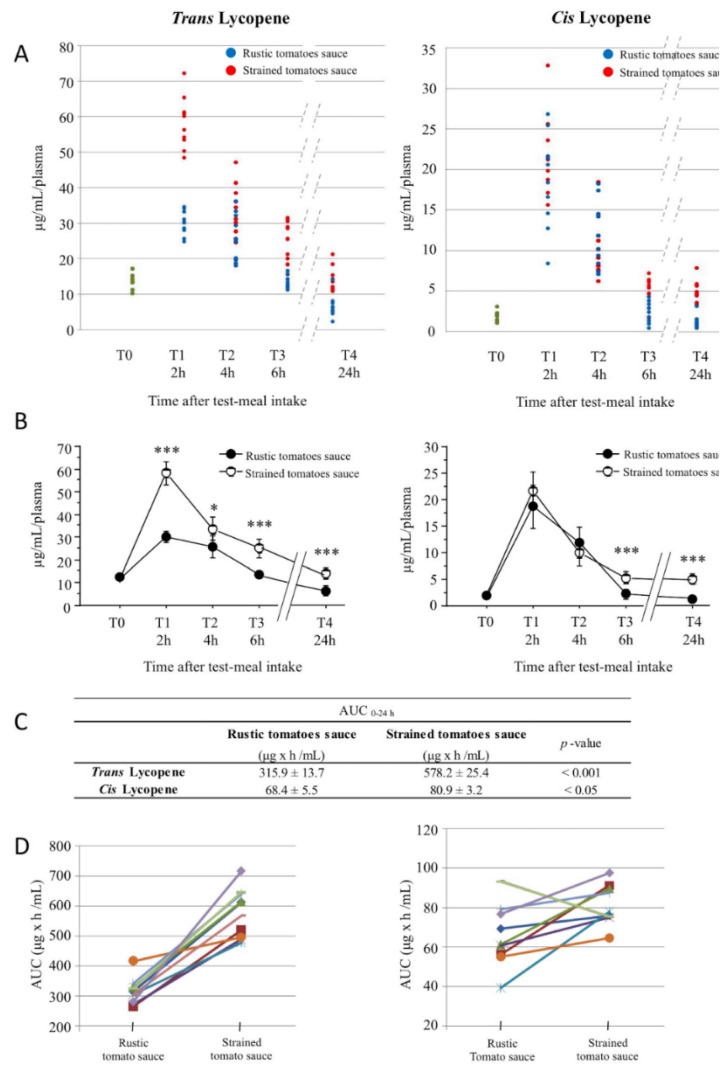
HPLC analysis of trans- and 5-*cis*-lycopene isoforms levels in plasma of the subjects after consumption of the day 1 test meal (acute-phase). (**A**) Dot plots indicate how the values relative to the single subjects are distributed according to the observation times. (**B**) The bars represent the 95% confidence intervals for the calculated averages * *p* < 0.05; *** *p* < 0.001. (**C**,**D**) The area under the curve over 24 h was derived using trapezoidal approximation. (**C**) The AUC values of *rustic tomato* sauce and *strained tomato* sauce were compared using a paired Student’s t-test and expressed as mean ± SD. (**D**) Line charts indicate how the values relative to the single subjects are modified depending on the type of sauce consumed.

In order to evaluate the effects of the two sauces consumption on lycopene plasma content, we determined the *cis*- and *trans*-lycopene plasma concentration using HPLC analyses in a time-course experiment 2, 4, 6, and 24 h after consumption of the test meal. Figure 2A shows the dot plots of the plasmatic *trans*- and *cis*-lycopene mean concentration (µg/mL/plasma) measured at basal level (T0; green points) and 2, 4, 6, and 24 h (T1, T2, T3, and T4) after the consumption of the test meal *rustic* tomatoes (blue points) or *strained tomatoes* (red points); for a more detailed description of data measurements, see Supplementary Table S1.

The *trans*- and *cis*-lycopene concentration values shown in Figure 2A are also reported as mean value ±SD for each time point in Figure 2B and Supplementary Table S2. *Cis*- and *trans*-lycopene plasma concentration had similar time-course profiles over 24 h in both sauces. In particular, the *trans*-lycopene concentration peeked at T1, 2 h after test meal consumption, and remained above the basal value (T0) up to 24 h in the plasma of subjects who consumed the *strained tomatoes* when compared to *rustic tomatoes* (Figure 2B, T1, T3, *p* < 0.001; T2 *p* < 0.05). No differences in lycopene plasma concentration were observed 2 and 4 h after the consumption of *strained tomatoes* compared to *rustic tomatoes* (Figure 2A). Conversely, significant differences were observed at T3 and T4 (Figure 2B, T3, T4 *p*< 0.001), 6 and 24 h after consumption of the test meal with *strained tomatoes* compared to *rustic tomatoes*, respectively. The area under the curve (AUC) at 0–24 h was derived using trapezoidal approximation (µg x h/mL). Subjects who consumed *strained tomatoes sauce* had a greater increase in both *trans*- and *cis*-lycopene concentrations than subjects who consumed *rustic tomatoes sauce* (Figure 2C *p* < 0.001 and *p* < 0.05, respectively). Finally, Figure 2D shows the AUC of the *trans*- and *cis*-lycopene concentrations obtained from each subject.

**Figure 3:**
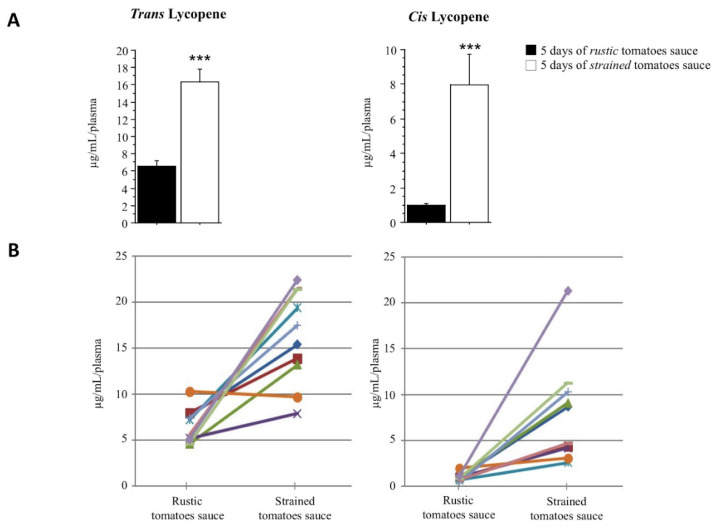
*Trans*- (left panel) and 5-*cis*- (right panel) lycopene isoforms were measured by HPLC in plasma of participants after 5 days of consumption of two different sauces. (**A**) Bars show the mean ± SD of plasma lycopene isoform concentration after 5 days of the consumption of *rustic tomatoes sauce* (black bars) or *strained tomatoes sauce* (white bars). *** *p* < 0.001. (**B**) Line charts indicate the plasma lycopene concentration of each subject (color code) according to the different sauce consumed.

We also determined the *trans*- and *cis*-lycopene plasma concentration by HPLC in subjects after consumption for 5 days of the test meal (chronic phase of experimental design, see Figure 1). Subjects who consumed the *strained tomatoes* sauce for 5 days had higher plasma concentrations of *trans*- and *cis*-lycopene than subjects who consumed the *rustic tomatoes* sauce (Figure 3A and Supplementary Table S3, compare white and black bars, respectively; *p*< 0.001). The data are reported as a line plot in Figure 3B.

### 3.3. Blood Antioxidant Potential Measured after Tomato Meal

**Figure 4:**
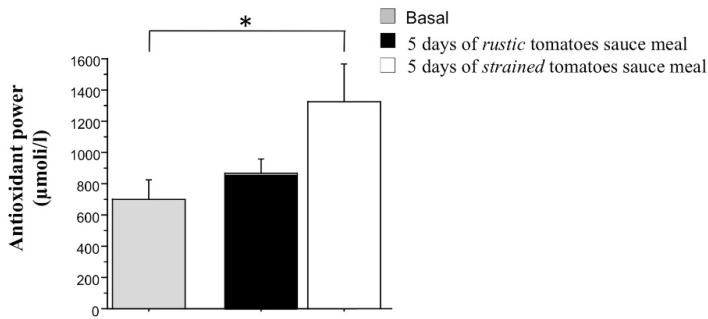
Biological antioxidant potential (BAP) detected as power associated to the ability of plasma barrier components to give reducing equivalents to reactive species in the plasma of subjects after 5 days consumption of the two sauces vs. basal BAP. Bars represent the mean ± SD of lycopene isoform concentration after 5 days of *rustic tomatoes sauce* (black bars) and *strained tomatoes sauce* (white bars) consumption vs. basal withdrawal in basal conditions * *p* < 0.05.

Since both *trans*- and *cis*-lycopene were found to be very low 2 h after the first meal (acute phase, ranging from 20 to 5 µg/mL/plasma for *trans*-lycopene and from 7 to 0 µg/mL/plasma for the *cis*-lycopene, respectively), we decided to not estimate BAP at the end of acute phase (1 day). In order to evaluate if 5 days of different sauces consumption gave an effect on the serum biological antioxidant power, we performed the BAP test assay at the end of the chronic phase (5 days). We found that the serum BAP result was positively affected, and it was statistically significant compared to the basal BAP value only in subjects who consumed the test meal prepared with strained tomatoes for 5 days (Figure 4 by comparing white to gray bar; *p*< 0.05).

## 4. Discussion

A fundamental aspect in nutrition is the knowledge of how the substances contained in the diet are modified and how, where, and to what extent they are absorbed by the human organism. These important aspects are known as “bioavailability” [44,45]. The exact knowledge about the bioavailability of each component of the diet is important for those naturally present in foods and also for those that are or should be added to the diet as supplements. The bioavailability of nutrients is a research area that has developed greatly in recent years, although human studies in this area are still limited [44,46].

Interestingly, the “way you cook” especially in terms of time and temperature is important for nutrients and also for their bioavailability, too. In fact, their content in food may be altered during cooking. Cooking food usually facilitates digestion and increases the absorption of many nutrients, but it is possible that some of them are reduced during these processes as for example vitamins and also mineral-containing compounds [47,48].

The final bioavailability of a nutrient (or food) may be defined as the sum of bioaccessibility and bioactivity, and it is the key point of nutritional efficiency [49].

**Figure 5:**
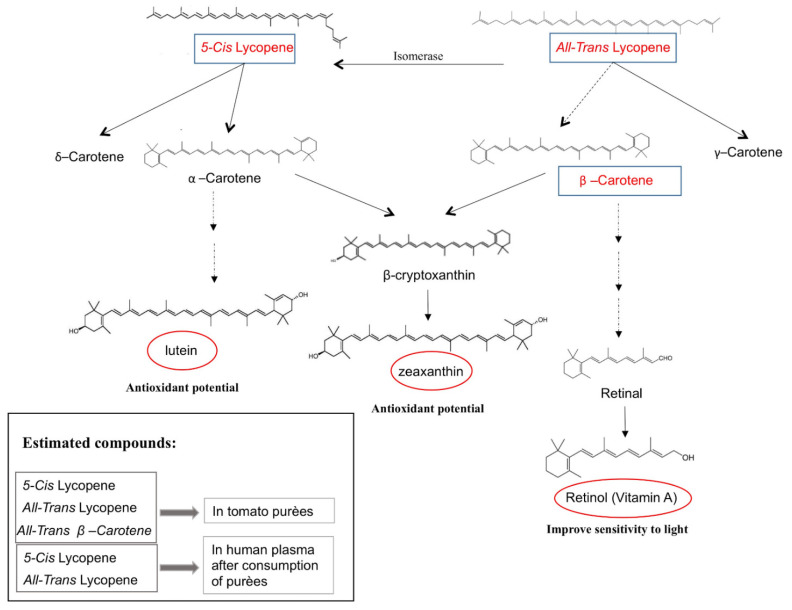
The biosynthesis pathway of carotenoids in humans. Solid and dashed arrows represent single and multiple enzymatic steps, respectively. The question mark indicates reactions not yet completely understood in humans. The estimated compounds are enclosed in rectangles while derivative final compounds for their beneficial effects in humans are in red oval circles.

Among these nutrients, carotenoids, which are present particularly in red/orange food [50], are metabolic precursors of vitamin A. Vitamin A, also known as retinol, is of the utmost importance for sight because, together with carotenoids, it is part of the components of rhodopsin, which is the substance present on the retina that gives the eye sensitivity to light (see Figure 5). More in detail, the product of light activation, metarhodopsin II, plays an important role in the visual phototransduction pathway. In humans, mutations in the *RHO* gene are associated to the onset of retinitis pigmentosa and to such other autosomal dominant genetic diseases as congenital stationary night blindness. In both pathologies, the eye fails to adapt to darkness, thereby resulting in a significantly reduced ability to see in dim light (vitamin A may improve this condition). Vitamin A is also useful for the development of bones and for their strengthening, for the growth of teeth and for its ability to provide and potentiate an immune response by the organism. Recent studies have shown that vitamin A alone has also anticancer properties [21,51,52,53].

The first aim of the present work was to assess the presence of these kind of compounds (lycopene isomers and β-carotene) in two different tomato pureés, after different boiling times with and without evo, and also to evaluate how they can be transformed once digested in the human body. We demonstrate that the boiling process enriched the concentrations of *cis*- and *trans*-lycopene in both the purées studied, and that this increase was further enhanced in the presence of evo during cooking (after various periods of boiling). Cooperstone et al. [31] compared the bioavailability of lycopene’s isomers in two different types of tomatoes (tangerine vs. red tomatoes), and they demonstrated the importance of this parameter and the relevance of this biological property.

We also found a significantly higher concentration of lycopene, both *cis*- and *trans*-isomers, in the blood plasma of volunteers after they had consumed the test-meal, particularly after the strained tomato purée intake. Furthermore, we demonstrated a statistically significant increase of lycopene plasma concentration only in subjects who consumed the strained tomatoes purée for 5 days. Interestingly, this increase seems to correlate with a significant increase of BAP evidenced only in the subjects that consumed the strained tomatoes for 5 days. The BAP test, which measures the reduction of ferric to ferrous ions, provides a reliable measure of the biological antioxidant potential of blood plasma. Antioxidants are groups of compounds that neutralize free radical and reactive oxygen species (ROS) in the cell, which have recently acquired an important role in counteracting the development of different diseases, such as cardiovascular and chronic-degenerative diseases, including cancer [53].

## 5. Conclusions

This study shows that such cooking processes as those described herein can increase the bioavailability of compounds contained in normally consumed food (such as tomatoes) and therefore their important biological processes, including those involved in disease-prevention mechanisms. Furthermore, differences in the commercial presentation of similar food nutrients (*rustic* or *strained* purée) may affect the nutritional benefit of tomatoes and derivative sauces, which constitute the principal component of the Mediterranean diet, which is a useful and necessary process to be taken into account in food processing.

In conclusion, we demonstrate that cooking time and the addition of olive oil improves the bioaccessibility of carotenoids in sauces made from commercial tomato purées, thereby improving antioxidant activity. Lastly, we show that this beneficial food may differ greatly in terms of carotenoid content and bioavailability for nutritional purposes.

## Figures and Tables

**Table 1:** 5-*cis*- and *trans*-lycopene isoforms and β-carotene content in tomato samples. “Puree” is uncooked tomato puree; “Boiled puree” is tomato puree boiled in a beaker for 10 min at 100 °C without evo; “Sauce” is tomato puree prepared with the addition of olive oil (50 g/L) and boiled for 20 min. Values represent the mean of three different measures ± SD. Fixed residue at 105 °C was 21.8% in *rustic* tomato purèe and 20.8% in the *strained* one.

	*Trans*-Lycopene (µg/g)		*Cis*-Lycopene (µg/g)		β-Carotene (µg/g)	
Shape	Rustic Tomatoes	Strained Tomatoes	Rustic Tomatoes	Strained Tomatoes	Rustic Tomatoes	Strained Tomatoes
Puree	249.6 ± 28.4	308.7 ± 27.3	16.6 ± 1.5	36.3 ± 2.4	7.6 ± 0.6	8.3 ± 1.1
Boiled puree	336.6 ± 28.6	324.8 ± 24.6	34.6 ± 2.0	40.3 ± 3.2	9.4 ± 0.9	10.5 ± 0.5
Sauce	321.8 ± 23.5	501.6 ± 14.5	32.2 ± 3.5	45.3 ± 3.2	14.1 ± 1.5	30.2 ± 2.8

**Table 2:** Effect of cooking time and evo addition on *trans* and *5-cis-lycopene* isoforms and β-carotene content in samples of sauces. Values represent the mean of three different measures ± SD.

	*Trans*-Lycopene (µg/g)				*Cis*-Lycopene (µg/g)				β-Carotene (µg/g)			
Cooking Time (min)	Rustic Tomatoes		Strained Tomatoes		Rustic Tomatoes		Strained Tomatoes		Rustic Tomatoes		Strained Tomatoes	
After Boiling	− evo	+ evo	− evo	+ evo	− evo	+ evo	− evo	+ evo	− evo	+ evo	− evo	+ evo
10	250.4 ± 15.9	316.6 ± 13.2	319.0 ± 22.0	468.8 ± 22.3	24.1 ± 1.1	28.4 ± 2.8	18.3 ± 0.5	32.2 ± 0.7	9.0 ± 1.0	12.4 ± 0.6	8.7 ± 0.3	20.4 ± 1.7
20	273.7 ± 16.5	324.5 ± 50.5	339.0 ± 29.2	489.6 ± 55.6	27.2 ± 2.0	30.1 ± 2.0	21.0 ± 1.8	35.8 ± 2.9	10.7 ± 0.8	15.4 ± 1.2	9.2 ± 0.3	25.0 ± 2.3
30	301.5 ± 38.6	330.7 ± 35.4	364.6 ± 29.1	525.7 ± 34.5	30.4 ± 0.9	34.8 ± 2.5	27.2 ± 2.4	40.6 ± 1.9	13.6 ± 1.4	18.4 ± 2.5	10.1 ± 0.6	34.9 ± 2.4
40	319.2 ± 13.4	343.3 ±27.7	410.3 ± 32.9	547.2 ± 51.6	36.6 ± 3.4	38.1 ± 4.4	32.6 ± 4.2	49.5 ± 4.6	15.0 ± 1.0	25.7 ± 1.1	12.4 ± 0.9	39.9 ± 1.7

## Supplementary Materials

The following are available online at https://www.mdpi.com/article/10.3390/nu13041336/s1, Table S1: Lycopene’s isoform estimation from T0 to T5; Table S2: *Trans*- and *Cis*-lycopene plasma concentration and Table S3: *Trans*- and *Cis*-lycopene plasma concentration in chronic phase consumption.
