# Supplementary material for: Nutritional Controlled Preparation and Administration of Different Tomato Purées Indicate Increase of β-Carotene and Lycopene Isoforms, and of Antioxidant Potential in Human Blood Bioavailability: A Pilot Study
Source: Nutrients. 2021 Apr 17;13(4):1336. doi: 10.3390/nu13041336 (PMC8073136; doi:10.3390/nu13041336)
Supplement: Supplementary file 1 [file nutrients-13-01336-s001.zip › Supplementary Material.pdf]

Nutritional Controlled Preparation and Administration of Different Tomato Purées Indicate Increase of β-Carotene and Lycopene Isoforms, and of Antioxidant Potential in Human Blood Bioavailability: A Pilot Study

Daniela Vitucci [1,†], Angela Amoresano [2,†], Marcella Nunziato [1,3,†], Simona Muoio [4], Andreina Alfieri [1,5], Giovannangelo Oriani [1], Luca Scalfi [6], Luigi Frusciante [7], Maria Manuela Rigano [7], Piero Pucci [1,2], Luigi Fontana [8], Pasqualina Buono [1,5,*] and Francesco Salvatore [1,3,*]

Table S1. **Lycopene's isoform estimation from T0 to T5.** The table shows the mean ± SD values for *trans*- and *cis*- lycopene concentrations analyzed in triplicate in baseline blood withdrawal at (T0) and following test-meal consumption at T1, T2, T3, T4 and after 5 days of sauces chronic consumption (T5).

	***Trans*- Lycopene (µg/mL/plasma)**											
**Subject**	**T0**		**T1**	**T2**	**T3**	**T4**	**T5**		**T1**	**T2**	**T3**	**T4**	**T5**
1	15.1 ± 0.9		31.1 ± 0.5	25.6 ± 0.3	13.4 ± 0.7	6.5 ± 0.7	7.6 ± 0.7		53.5 ± 0.9	34.5 ± 1.1	31.2 ± 0.9	12.1 ± 0.6	15.4 ± 0.5
2	13.7 ± 0.6		25.6 ± 0.7	19.6 ± 0.5	11.7 ± 1.7	5.4 ± 1.2	8.0 ± 1.2		60.8 ± 0.4	25.6 ± 0.6	21.3 ± 1.1	13.5 ± 0.8	13.9 ± 1.0
3	10.3 ± 1.2		34.6 ± 0.6	20.2 ± 2.0	14.3 ± 0.5	7.6 ± 0.6	4.6 ± 0.5		65.4 ± 0.7	27.7 ± 0.3	28.9 ± 0.4	14.3 ± 0.9	13.2 ± 0.4
4	14.3 ± 0.6		24.9 ± 0.7	18.1 ± 1.3	12.0 ± 0.7	6.2 ± 1.7	5.2 ± 1.3		60.2 ± 0.5	24.6 ± 0.9	20.1 ± 2.2	11.6 ± 1.0	7.9 ± 0.9
5	17.2 ± 0.8	Rustic Tomatoes sauce	28.1 ± 2.3	18.8 ± 2.0	12.7 ± 1.1	8.1 ± 1.4	7.2 ± 1.0	Strained Tomatoes sauce	72.1 ± 0.7	31.2 ± 1.4	15.6 ± 0.8	10.9 ± 0.6	19.4 ± 0.5
6	13.4 ± 1.0		30.3 ± 1.0	24.9 ± 1.5	16.6 ± 0.8	14.1 ± 1.2	10.3 ± 1.0		61.2 ± 0.4	30.2 ± 0.6	18.4 ± 0.5	12.1 ± 0.8	9.7 ± 0.7
7	11.3 ± 1.2		33.2 ± 1.0	29.4 ± 1.0	15.6 ± 0.6	5.4 ± 0.8	7.4 ± 0.8		54.1 ± 0.1	36.1 ± 1.2	30.6 ± 0.6	15.4 ± 0.4	17.5 ± 0.3
8	15.2 ± 1.5		34.1 ± 1.5	32.1 ± 3.1	11.2 ± 1.9	5.1 ± 2.9	5.6 ± 0.6		56.3 ± 0.4	38.5 ± 0.4	25.6 ± 0.4	11.3 ± 0.4	21.5 ± 0.7
9	10.3 ± 0.9		30.2 ± 1.4	36.2 ± 1.0	14.2 ± 1.3	4.6 ± 0.7	4.6 ± 0.5		50.3 ± 0.7	41.3 ± 0.6	28.6 ± 0.4	18.4 ± 0.3	21.4 ± 0.3
10	13.3 ± 0.6		28.6 ± 1.3	33.4 ± 1.0	12.3 ± 1.1	2.3 ± 1.1	5.1 ± 1.0		48.4 ± 0.4	47.1 ± 0.6	31.5 ± 0.3	21.3 ± 0.5	22.4 ± 0.4

	***Cis*- Lycopene (µg/mL/plasma)**											
**Subject**	**T0**		**T1**	**T2**	**T3**	**T4**	**T5**		**T1**	**T2**	**T3**	**T4**	**T5**
1	2.1 ± 0.7		20.6 ± 0.6	11.9 ± 0.2	1.3 ± 0.7	0.5 ± 0.3	1.0 ± 0.7		21.2 ± 1.5	8.1 ± 1.2	4.7 ± 0.6	5.9 ± 0.3	8.7 ± 1.7
2	1.9 ± 0.4		16.6 ± 0.5	7.4 ± 0.7	3.4 ± 0.9	0.8 ± 0.4	1.0 ± 0.7		32.8 ± 0.7	6.2 ± 1.1	1.7 ± 0.4	7.9 ± 0.6	4.4 ± 0.8
3	1.5 ± 0.4		18.4 ± 1.1	9.4 ± 1.1	1.8 ± 0.6	0.6 ± 0.4	1.0 ± 0.8		25.6 ± 1.2	9.1 ± 0.8	6.4 ± 0.7	5.7 ± 0.5	9.1 ± 0.6
4	2.1 ± 0.4		14.6 ± 1.0	10.2 ± 1.8	4.3 ± 1.1	1.3 ± 0.7	1.0 ± 1.0		21.6 ± 1.2	8.1 ± 0.5	4.3 ± 0.8	4.9 ± 0.9	4.2 ± 0.3
5	2.3 ± 0.5	Rustic Tomatoes sauce	8.4 ± 0.8	7.1 ± 1.2	2.9 ± 0.6	0.8 ± 0.4	0.7 ± 0.6	Strained Tomatoes sauce	17.1 ± 0.8	11.8 ± 1.0	7.2 ± 0.7	3.4 ± 1.2	2.6 ± 0.4
6	2.1 ± 1.6		12.7 ± 0.8	8.4 ± 0.9	3.8 ± 0.4	3.2 ± 1.2	2.0 ± 1.3		15.6 ± 0.4	7.6 ± 0.8	6.1 ± 0.7	4.6 ± 0.7	3.1 ± 0.4
7	1.4 ± 1.0		21.6 ± 0.8	14.2 ± 1.2	2.4 ± 0.8	1.2 ± 0.7	0.5 ± 0.3		23.6 ± 0.6	10.2 ± 0.6	6.4 ± 0.7	4.6 ± 0.3	10.3 ± 0.2
8	1.1 ± 0.6		26.8 ± 1.5	17.4 ± 1.1	1.3 ± 0.9	1.1 ± 0.2	0.7 ± 0.4		19.8 ± 0.4	8.4 ± 0.4	5.7 ± 0.5	5.9 ± 0.3	4.7 ± 0.5
9	2.3 ± 0.9		25.4 ± 0.6	18.2 ± 1.5	1.0 ± 0.5	1.6 ± 0.5	0.9 ± 0.2		18.7 ± 0.5	11.2 ± 0.8	5.4 ± 0.4	3.6 ± 0.3	11.3 ± 0.3
10	3.1 ± 1.2		21.4 ± 0.9	14.5 ± 0.7	0.5 ± 0.2	1.0 ± 0.6	1.1 ± 0.9		21.6 ± 0.3	18.4 ± 0.3	4.7 ± 0.5	4.5 ± 0.3	21.3 ± 0.3

Table S2. ***Trans*- and *Cis*-lycopene plasma concentration.** Time-course (mean ± DS value) of *trans*- and *cis*-lycopene plasma concentration after meal consumption (see Fig. 2B).

	Time after meal intake	Rustic tomatoes sauce	Strained tomatoes sauce	*p* -value
***Trans* - Lycopene (µg/mL/plasma)**	T1 2h	30.1 ± 3.3	58.2 ± 7.2	< 0.001
	T2 4h	25.8 ± 6.6	33.7 ± 7.2	< 0.05
	T3 6h	13.4 ± 1.8	25.2 ± 5.9	< 0.001
	T4 24h	6.5 ± 3.1	14.1 ± 3.4	< 0.001
***Cis* - Lycopene (µg/mL/plasma)**	T1 2h	21.7 ± 4.9	18.6 ± 5.7	> 0.05
	T2 4h	10.0 ± 3.4	11.9 ± 4.0	> 0.05
	T3 6h	5.3 ± 1.5	2.3 ± 1.3	< 0.001
	T4 24h	5.1 ± 1.5	1.2 ± 0.8	< 0.001

Table S3. ***Trans*- and *Cis*-lycopene plasma concentration in chronic phase consumption.** Mean values ± DS of *trans*- and *cis*- plasma lycopene concentration after 5 days consumption of two different sauces (see Figure 3 A).

	Time after meal intake	Rustic tomatoes sauce	Strained tomatoes sauce	*p* -value
***Trans* - Lycopene (µg/mL/plasma)**	T5	6.6 ± 1.8	16.2 ± 5.1	< 0.001
***Cis* - Lycopene (µg/mL/plasma)**	T5	1.0 ± 0.4	8.0 ± 5.6	< 0.001
